# Coping with multimorbidity in old age – a qualitative study

**DOI:** 10.1186/1471-2296-13-45

**Published:** 2012-05-29

**Authors:** Christin Löffler, Hanna Kaduszkiewicz, Carl-Otto Stolzenbach, Waldemar Streich, Angela Fuchs, Hendrik van den Bussche, Friederike Stolper, Attila Altiner

**Affiliations:** 1 Institute of General Practice, Universitätsmedizin Rostock, Doberaner Str. 142, Rostock, 18057, Germany; 2 Department of Primary Medical Care, Universitätsklinikum Hamburg-Eppendorf, Martinistr. 52, Hamburg, 20246, Germany; 3 Department of General Practice, Universitätsklinikum Düsseldorf, Moorenstr. 5, Düsseldorf, 40225, Germany

**Keywords:** Coping, Multimorbidity, Primary care, Qualitative research

## Abstract

**Background:**

Comparatively few studies address the problems related to multimorbidity. This is surprising, since multimorbidity is a particular challenge for both general practitioners and patients. This study focuses on the latter, analyzing the way patients aged 65–85 cope with multimorbidity.

**Methods:**

19 narrative in-depth interviews with multimorbid patients were conducted. The data was analysed using grounded theory. Of the 19 interviewed patients 13 were female and 6 male. Mean age was 75 years. Participating patients showed a relatively homogeneous socio-economic status. Patients were recruited from the German city of Hamburg and the state of North Rhine-Westphalia.

**Results:**

Despite suffering from multimorbidity, interviewees held positive attitudes towards life: At the social level, patients tried to preserve their autonomy to the most possible extent. At the emotional level, interviewees oscillated between anxiety and strength - having, however, a positive approach to life. At the practical level, patients aimed at keeping their diseases under control. The patients tended to be critical in regards to medication.

**Conclusions:**

These findings might have implications for the treatment of multimorbid patients in primary care and further research: The generally presumed passivity of older individuals towards medical treatment, which can be found in literature, is not evident among our sample of older patients. In future, treatment of these patients might take their potential for pro-active cooperation more strongly into account than it is currently the case.

## Background

Today, multimorbidity is a common problem among the elderly and its occurrence rises with age [1-3]. Research has shown that in Europe, for instance, more than 60% of people aged 65+ can be classified as multimorbid [4,5]. Figures for the US show a similar pattern [6,7]. Whereas numerous studies focus on chronic conditions – both from the physicians’ as well as the patients’ point of view – few studies address multimorbidity. This is rather surprising, since multimorbidity is a particular challenge – not only for General Practitioners (GPs), but also for patients. In 2005, Boyd and colleagues, for instance, reviewed clinical practice guidelines for the 15 most common chronic diseases in the US. They conclude that strict adherence to current guidelines when caring for older people with multimorbidity may cause severe undesirable effects including adverse reactions between drugs and diseases [8].

As there is often no effective cure for chronic conditions and multimorbidity, the principal aims of medical treatment are the secondary prevention of complications and the improvement of functional capacity and quality of life [9,10]. Studies show that the availability of coping resources has a major impact on the way patients handle their chronic conditions [11-17]. According to Lazarus and Folkman, coping refers to the "constantly changing cognitive and behavioral efforts to manage specific external and/or internal demands that are appraised as taxing or exceeding the resources of the person" [18]. In contrast to previous research, Lazarus and Folkman define coping as a *process* that is concerned with what a person within a specific context thinks or does. The authors distinguish between *emotion-focused forms of coping* and *problem-focused forms of coping*. Whereas the former are likely to occur whenever individuals appraise that nothing can be done to modify “harmful, threatening, or challenging environmental conditions” [19], problem-focused forms of coping are possible when individuals assume that the prevailing conditions might be changed. Lazarus and Folkman argue that coping is a process that evolves from resources. Among others they identified the following major categories of coping resources: health and energy, positive beliefs, problem-solving skills, social skills, social support and material resources. Also, coping resources are assumed to be multidimensional. However, the utilization of available coping resources might be constrained by several factors, such as personal or environmental constraints or the level of threat [18].

Several studies investigate the way adults cope with specific chronic diseases [12,20-22]. Charmaz, for instance, explores the suffering of chronically ill patients. She describes the loss of self in these people “who observe their former self-images crumbling away without the simultaneous development of equally valued new ones” [23]. Corbin and Strauss investigate how chronic illness is managed at home and the impact it has upon patient and spouse [24]. More recent studies point to the importance of social support and social networks [25-27] and the enhancement of self-efficacy [18,28] and self-management [29,30].

In the present paper, we focus on an issue that has so far remained widely unattended: coping with multimorbidity in old age. In particular, our research question is: How do old aged multimorbid patients cope with their multiple chronic diseases?

## Methods

### Design and setting

Employing a qualitative research design, we conducted narrative in-depth interviews with a total of 19 multimorbid patients. Narrative interviews are characterized by an initial phase of topic formulation carried out by the interviewer, followed by the main narrative phase. During this phase, the interviewer takes notes. When the interviewee stops talking, the interviewer refers to the information he gained so far and stimulates the interviewee to go into details. Also, external open-ended questions, formulated prior to the interview, were asked [31]. We included participants from the German city of Hamburg and several urban and rural areas within North Rhine-Westphalia. The interviews have an average length of 1 hour.

### Patient recruitment

Field work took place between November 2008 and March 2009. As far as sampling was concerned, 2 samples were compiled: Local GPs were contacted and recruited by the Department of Primary Medical Care at the University Medical Center Hamburg-Eppendorf and the Department of General Practice at the University of Duesseldorf. We included 2 patients per GP. Patient inclusion criteria were: aged 65–85; a minimum of 3 chronic conditions including at least one musculoskeletal disorder; and at least one visit to the GP within the last three months. Although multimorbidity usually refers to 2 or more chronic conditions, we opted for a narrowed definition as we intended only to include those patients who experienced a certain level of health related limitations and restrictions. Participating GPs were asked to generate a register containing all interviewees meeting these criteria; eligible patients were chosen randomly by GPs to be invited for the interview. All invited patients agreed to be interviewed. The study was approved by the Ethics Committee of the Chamber of Physicians of Hamburg (reference number PV3091).

### Sample description

After 17 interviews data saturation was reached. Here, data saturation refers to the point at which additional interviews do not provide any new insights into the topic [32]. Since further appointments were already fixed, we realized another 2 interviews. Of all 19 interviewed patients 13 were female and 6 male. The mean age of the interviewees was 75 years. The participating patients showed a relatively homogeneous socio-economic status: 8 patients had completed lower secondary education (Germ. “Volksschule”), but no vocational training (Germ. “Lehre”), and 11 patients had successfully completed vocational training. Most participants were married, 2 were divorced, and 6 widowed. Their number of children ranged from 0 to 5, with a median of 2 children. Mainly, participating patients suffered from hypertension, coronary heart disease, congestive heart failure, diabetes mellitus type 2, COPD, arterial fibrillation, coxarthrosis, gonarthrosis, lumbar spinal stenosis, and major depression. Patients had known and had been treated by their GP ranging from 5 to 20 years, with a mean of 13 years (Table 1).

**Table 1:** Characteristics of the 19 multimorbid patients interviewed for the study

	Number of patients
**Gender**	
Female	13
Male	6
**Age**	
65–69 years	4
70–74 years	2
75–79 years	8
80–85 years	5
*Mean age*	*75 years*
**Marital status**	
Married	11
Divorced	2
Widowed	6
**Number of children**	
0	2
1	6
2	6
3	2
4	2
5	1
*Mean number of children*	*2*
**Education**	
No degree	4
Lower secondary (,,Volksschule“)	11
Higher secondary (,,Mittlere Reife“)	4
**Vocational training**	
Yes	11
No	8
**Active at the labour market during lifetime**	
Yes	19
**Years treated by current GP**	
0–5 years	1
6–10 years	5
11–15 years	8
More than 15 years	5
*Mean years*	*13 years*

### Data analysis

Fieldwork was carried out by WS, COS, HK and Carsten A. Reich. All interviews were audio taped and transcribed verbatim. Since the authors of this paper belong to a variety of disciplines (e.g. family medicine, sociology, health care research), the analyses benefited from a multidisciplinary perspective: The authors drafted memos and discussed them regularly. We applied the constant comparative method of analysis from *grounded theory*[32-34] to the data by employing the three steps of coding and categorizing: *open codingaxial coding,* and *selective coding.* Open coding refers to labeling of data, sentence-by-sentence or paragraph-by-paragraph. Next, labels are combined into categories, and axes between them are identified. During axial coding, the number of codes is reduced and the different axes between the phenomenon and its context, intervening factors, and consequences are constructed. Finally, selective coding aims at “elaborat[ing] the core category around which the other developed categories can be grouped and by which they are integrated” [35]. With respect to sampling procedure, the study deviates from *grounded theory*: Instead of theoretical sampling, we opted for a randomized sampling procedure to reduce selection bias caused by GPs’ preferences for including certain patients while disregarding e.g. patients perceived as being difficult. Coding and categorising of the interviews was done by CL and FS and discussed with the other researchers continuously in the process of the analysis. All data was managed and coded using a qualitative data software programme (*QSR NVivo version 8*). After categorising all codes referring to coping with multimorbidity 3 major categories emerged. These categories will be presented in the following section. The quotes used to illustrate our results were translated by a professional bilingual translator.

## Results

Consequences of multimorbidity for everyday life proved to be as manifold as multimorbidity itself: For instance, interviewees were restricted in their daily routine through fatigue, shortness of breath, limited mobility, and anxiety. The consequences went beyond physical symptoms and had an impact on many spheres of life. Our analyses showed that interviewees dealt with multimorbidity mainly at 3 levels: at a social, an emotional and a practical level.

### Coping at the social level

Coping at the social level refers to patients’ effort in conducting and holding up a meaningful life and in keeping their autonomy. Patients with moderate limitations tried to keep their social role to the most possible extent. In order to show this pattern, in the following we present some quotes. A typical example of the described approach is Mrs. A. Being aged 83 and suffering from atrial fibrillation, rheumatism, hypertension, and gonarthrosis she resided with her husband and one of her adult children in a house. Despite painful disorders, Mrs. A continued to take major care of the household, family meals, and the garden. She describes her and her husband’s daily routines as follows:

"“Every day, we've got our work, right? We know exactly what we need to do. But what we also know is that we're done by lunch, right? We get up at 7.30/7.15 that's when we get up. We have breakfast at eight. My husband prepares breakfast every morning. That's the time I get ready. So, it's 8.30 by the time we're ready. But then we work all morning until lunchtime. Then cooking lunch needs to be done. And when we're finished cleaning the kitchen it's already 1 o'clock. Then we call it a day and take our break.“"

Other multimorbid patients used different strategies for sustaining a meaningful life: they adopted a dog, they searched for new hobbies, or they were creative in continuing to do sports. Mr. B is an example of this kind. He was aged 74 and suffered from coronary heart disease, type 2 diabetes and joint disorders. Since he was not able to keep his balance anymore, Mr. B felt limited in his capacity to ride a bike. He came up with a solution himself: instead of using a bike, he bought a tricycle. This way he continued to keep his mobility – for this patient an essential part of his social life.

Patients with less mobility and suffering from major restrictions focused primarily on preserving their autonomy. A characteristic example is Mrs. C. She was aged 82 and had lived through a long history of serious surgeries and diseases. At the time of the interview she was suffering most from chronic back pain. In spite of difficulties in handling everyday routines she rejected her GPs offer to file an application for nursing allowances.^a^ When talking about that offer, she trivialized her health situation and emphasized her self-determined choice of whom to ask for help:

"“So in the evenings I ask my son-in-law: Could you peel a couple of potatoes for tomorrow? Could you open the lid on this jar and unscrew the cap of this water bottle? Do you know how hard that is? […] I tell you, the diseases are all bearable. Even if I can’t comb my own hair – well, that’s not so bad. I get my hair combed in the mornings so now my doctor suggests that we apply for nursing allowance. So I tell her, ‘No, why should we? I’m still able to talk; I can still talk to other people. And everything else I can do by myself. No,’ I tell her, ‘It’s not that bad yet. I really don’t need any nursing allowance.’ […] Good thing I have a good family, right?“"

In general, patients were characterized by a “*can-do-approach to life*”: They activated all their resources for the continuation of a meaningful and autonomous life.

However, some patients faced stronger barriers in coping with the social dimension of their situation. This was particularly true for patients suffering from uncommon disorders or from diseases people could not relate to, e.g. vegetative problems. A distinctive case is Mrs. D. At the age of 63 she was afflicted – among other disorders – with food intolerance. She felt abandoned by her social surrounding:

"“Should I be straightforward with you?! You’re left all to yourself. (pause) You’re all alone. It’s, well, the other people listen to your problem but basically they don’t really know how to deal with it and some don’t even want to. Even within the family – they know I’ve got this problem and then later we don’t even talk ‘bout it. […]“"

### Coping at the emotional level

This category includes patients’ ways of dealing emotionally with the challenges involved in multimorbidity. Interviewees experienced an interplay of anxiety, desperation, and dolefulness on the one side and strength and euphoria on the other side. Patients’ quality of life was highly influenced by the way patients coped with their multiple chronic conditions. A typical example of hopelessness and desperation is Mrs. E. She was aged 77 and suffering from arthritis, COPD, and vascular obliteration of one eye. The latter was the reason for fearing the loss of her eyesight:

"“[I’m worried] that I might not be able to do anything here anymore, that’s … my son, he was so sad. (crying, pause) When he was here with me last week, I told him, well, that when I can’t see him anymore, please not have me go to a nursing home. I really only want to be here and maybe that’s… My partner also knows about it and I told him that, don’t you dare leave me anywhere by myself, that’s horrible. That’s what I fear most – but I keep trying to face it, really (speaking sobbingly)”"

Another patient, Mr. F, aged 69, reported some kind of calming down during the period of sustaining a serious atrioventricular block and receiving a cardiac pacemaker:

"“However, you suddenly become very calm and very serene. I felt instinctively that, if this isn’t fixed, right, you’ll die, you know? And then I also thought, well, this guy that they nailed to the cross, right – I’ll lay my fate in his hands. There’s nothing more you can do then – nobody can.”"

After recovery, Mr. F put an effort into actualizing his desires and dreams: He arranged holidays at the Mediterranean Sea and flying with a hot air balloon. This “*positive approach to life”* was also evident in the interview with Mrs. G. Being aged 75 and treated for aortic valve calcification, hypertension, and musculoskeletal disorders. She stated, “there are people that are much, much worse off. That’s what I always keep in mind.” Daily mastering the difficulties of chronic diseases and multimorbidity without capitulating was the prevailing pattern among interviewees. This was also true for Mrs. H. Having experienced several serious disorders and surgeries, at the age of 83 Mrs. H suffered a herniated disc. She remembered the effort she put into living a normal life again:

"“But basically I can feel as lousy as may be – I simply don’t give up. Last year it got so bad, I had to pull myself up the stairs. I really wasn’t able to place one foot in front of the other. And I always kept thinking, ‘Lord almighty, is this the way you’re supposed to end?’ But as it kept getting better, I used the stairs as a kind of exercise. So I sometimes went up and down those stairs 25 times a day. But, you know, I did it.”"

### Coping at the practical level

Coping at the practical level refers to patients’ efforts in dealing with their multiple chronic diseases from a practical point of view, including management of physical examinations, therapies, medication and so forth. Interviewees looked very intensively into the subject of treating and medicating their conditions. Also, they put much effort into “*keeping their diseases under control*”. Patients reported, for instance, about storing and comparing blood values. Mr. I is a typical example of this pattern. He was aged 77 and treated for type 2 diabetes, hypertension, and gout. His wife, who was present during the interview, stated the following:

"“These checkups every three months … that’s good for everyone. It’s good for us because we control it. We get to take the results home with us. Whenever we ask for it at the doctor’s office, we get a copy of the blood test results, right? Then we compare them with the last time and in case something is wrong, then we somewhat try to adjust, if possible.“"

In general, patients were well informed about their diseases and disorders. They used to seek advice from books, journals, pharmacies, or the internet. A patient suffering, among other disorders, from migraine stated:

"“I read a lot of magazines and newspapers and quite often they include reports about migraine and I know 100% certain how I need to react.”; “I read a lot about my sickness in books.”."

Some patients turned to their GP whenever they had questions or doubts. Nonetheless, patients believed that you need to help yourself, otherwise “nobody helps” and “you are lost”. This was the case also for Mr. J. Being aged 74 and suffering from coronary heart disease, type 2 diabetes, and osteoarthritis he believed:

"“And that proves once again that you need to listen to your own body and decide all for yourself. What is good for me? And if I didn’t do that, no doctor would be able to help me.”"

As far as adherence to lifestyle recommendations was concerned, almost all patients stated to stick to the general advice of their GPs. Patients were aware of and complied with dietary needs and exercised. However, with respect to medication, patients showed a lower level of adherence. They tended to be rather critical and emphasized the dual nature of drugs: Mitigating symptoms and improving quality of life on the one side and leading to adverse reactions and new symptoms on the other side. Mrs. K is a typical example of a patient who is critical towards medication. At the same time, however, she prioritizes which pills are necessary to take and which not. She was aged 75 and treated for type 2 diabetes, arthritis, gastro-esophageal reflux disease, and hypertension. She reported:

"“That’s some really strong stuff, these diabetes pills, but I know I need to take them. And what I always umh, that was this [pill] the urologist gave me, like I said, but they go right to your stomach, so I didn’t take them, because I, I told myself, and my conscience told me, leave them, they make you sick. You see, it’s an antipathy that I’ve got against these pills. I’ve never got a clear conscience when I take something. I’m even really sad, now that I have to treat my diabetes like that. I also don’t take sleeping pills. I’d lie awake for three hours rather […] I’d have a really guilty conscience sneaking through life on sleeping pills.”"

A similar view was held by Mrs. L, aged 69 and suffering from COPD and several musculoskeletal disorders. She ignored her GPs recommendation to vary her daily amount of cortisone and continued to take the same little dosage.

"“Yeah, she [her GP] reckoned I'd feel better if I increased the dosage. But I'd rather suffer two or three days – then I've got it under control again.”"

The belief that drugs would impair health as a matter of principle recurred among our interviewees: A 78 years old female patient suffering from gonarthrosis, metabolic disorder, depression, and chronic cough stated: “And if you just keep taking those pills (…) all you do is harm yourself“; “I'm not a big fan of this constant pill-taking, because my stomach can't handle it.”

Given the age of the interviewed patients, the trend towards controlling existing diseases, informing oneself actively about disorders, and being critical about medication is rather surprising: Interviewees had much less paternalistic expectations toward their GP than one would expect.

**Figure 1:**
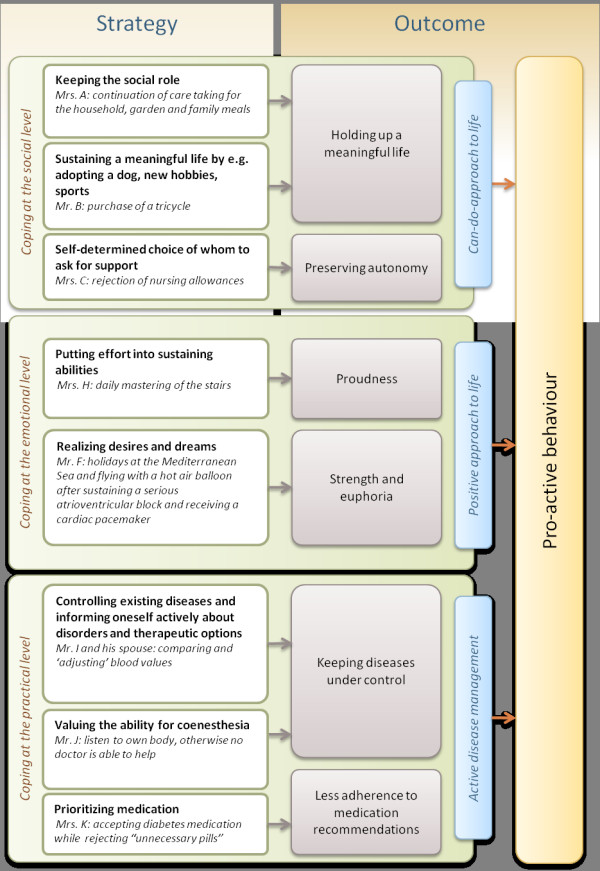
Coping categories, strategies, and outcomes among multimorbid old aged patients derived from 19 narrative in-depth interviews.

In Figure 1 we summarize the three identified coping categories by emphasizing the coping strategies of the interviewed patients, the respective outcomes and by referring to the examples described in the paper. Despite the identification of patients who faced strong problems in coping with multimorbidity, the majority of interviewees had a “can-do-approach” and a positive approach to life, resulting in a pro-active behaviour.

## Discussion

This paper focuses on the question, how old aged multimorbid patients cope with their multiple chronic diseases. The interviews provide evidence that despite multimorbidity interviewees held positive attitudes towards life: At the social level, patients tried to keep their social role and to preserve their autonomy to the most possible extent. This was evident in their “*can-do approach to life*”. At the emotional level, interviewees oscillated between anxiety and strength – having, however, a “*positive approach to life*”. And finally, at the practical level, patients aimed at “*keeping their diseases under control*”. Astonishingly, the patients we interviewed were not part of an intellectual elite, but belonged to the middle class and represented ‘ordinary people’. Also, patients were concordant with their GPs’ recommendations concerning lifestyle changes, complied with dietary needs and exercised. However, patients tended to be critical in regards to medication and emphasized the dual nature of drugs: Mitigating symptoms and improving quality of life on the one side, and leading to adverse reactions and new symptoms on the other side. This finding supports Horne and Weinman’s framework on the relationship between *necessity* of medication and *concerns* related to medication. Conducting a cross-sectional study, the authors found that higher necessity scores correlate with higher reported adherence, whereas higher concerns correlate with lower reported adherence [36]. The fact that multimorbid patients reported not adhering to medication recommendations highlights one important aspect of the set of problems related to polypharmacy: As Boyd and colleagues emphasized, referring to current clinical practice guidelines for single chronic diseases, the pharmaceutical management of multimorbid patients is hardly manageable, neither for physicians nor for patients [8].

Our findings give some evidence that today’s generation of seventy-year-old people might be different from previous generations of this age: Literature shows that earlier generations of older adults were less likely than others to cope by information seeking or by engaging in wish fulfilling fantasies [37,38]. Our interviewees, though, were *pro-active* in seeking information, adhering to GP’s lifestyle recommendations and questioning the benefit of drugs. Further research is necessary to evaluate whether this pro-active behaviour is an emerging phenomenon and if so, how it relates to relevant outcomes e.g. quality of life. One might suggest, that pro-active behaviour is related to increasing levels of life satisfaction and quality of life.

With respect to existing approaches on coping, the emerging categories of coping at the social, emotional and practical level might be integrated into Lazarus and Folkman’s differentiation between emotion-focused forms of coping and problem-focused forms of coping. Both frameworks might be understood as one dimension in a diagram, where coping at each level might be distinguished with respect to emotion-focused forms and problem-focused forms of coping. Among our interviewees, we find both forms of coping in all of the three categories. For instance, at the social level Mr. B employed a problem-focused form of coping by purchasing a tricycle. Mrs. D, though, used an emotion-focused form of coping: She accepted that people are indifferent towards her food intolerance and all problems related to that.

The study goes beyond previous studies on disease coping as we focus on multimorbidity instead of a single chronic disease. The active disease management that we found among interviewees might be highly related to multimorbidity: Since the co-existence of multiple chronic diseases requires careful and coordinated treatment and care, patients might have learnt to put priorities with regard to their health, and to act actively.

As far as limitations are concerned, our study is not based upon theoretical sampling as mentioned above, but upon 2 randomized samples, which were compiled in two different regions. After the first interviews were discussed within the team of researchers, we decided to further pursue this path as our data included cases of high contrast. Nonetheless, this procedure does not allow for a completely theory-driven approach of data generation and analysis as it is aimed at by grounded theory.

## Conclusions

These findings might have implications for the treatment of multimorbid and chronically ill patients in primary care: Our data suggests that the generally presumed passivity of older individuals towards medical treatment might no longer be prevalent in today’s generation of older patients. In future, treatment of these patients might take their potential for pro-active cooperation more strongly into account than it is currently the case.

However, in order to do so, patients will require possibilities to express their emotions and concerns. GPs, on the other side, will need concepts that allow for identifying the different resources patients have. Future research might follow this path by developing concepts that allow for the integration of patients’ pro-active attitudes and behaviour. Our findings support the chronic care model [39,40] with its focus on self-management and decision support. Applying the chronic care model for older patients with multimorbidity may serve as a framework to foster pro-active coping. Furthermore future Disease Management Programmes for multimorbid patients might include specific interventions to empower and address patients’ resources for pro-active coping with multimorbidity.

## Endnotes

^a^ In Germany, nursing allowance is a social benefit for patients in need of care. Since 1995, nursing care insurance is statutory. Depending on the patient’s level of nursing care dependency, nursing allowances for care-giving expenses are remitted.

## Competing interests

The authors declare that they have no competing interest.

## Authors’ contributions

HK and AA initiated and designed the study. Fieldwork was carried out by WS, COS, HK and Carsten A. Reich. Coding and categorising of the interviews was done by CL and FS and discussed with all authors continuously in the process of the analysis. All authors drafted memos and discussed them regularly. The paper was drafted by CL. All authors read and approved the final manuscript.

## Pre-publication history

The pre-publication history for this paper can be accessed here:

http://www.biomedcentral.com/1471-2296/13/45/prepub
